# 189 Development of the Youth-Physical Activity Towards Health in Northern Ireland (Y-PATH NI) Study

**DOI:** 10.1093/eurpub/ckae114.160

**Published:** 2024-09-26

**Authors:** Angela Carlin, Julia McClelland, Alison Gallagher, Sarahjane Belton, Wesley O’Brien, Marie Murphy

**Affiliations:** Centre for Exercise Medicine, Physical Activity and Health, Sports and Exercise Sciences Research Ins, Ulster University, Belfast, United Kingdom; Centre for Exercise Medicine, Physical Activity and Health, Sports and Exercise Sciences Research Ins, Ulster University, Belfast, United Kingdom; Nutrition Innovation Centre for Food and Health (NICHE), Biomedical Sciences Research Institute, Ulster University, Coleraine, United Kingdom; School of Health and Human Performance, Dublin City University, Dublin, Ireland; School of Education, University College Cork, Cork, Ireland; Centre for Exercise Medicine, Physical Activity and Health, Sports and Exercise Sciences Research Ins, Ulster University, Belfast, United Kingdom; Physical Activity for Health Research Centre (PHARC), Institute for Sport, Physical Education and Health Sciences, University of Edinburgh, Edinburgh, Scotland

## Abstract

**Purpose:**

The Youth-Physical Activity Towards Health (Y-PATH) programme is an evidence-based physical literacy intervention, aimed at enhancing provision of physical education (PE). Previous research has demonstrated the effectiveness of Y-PATH at increasing children’s physical activity levels and fundamental movement skills in Ireland. As Northern Ireland (NI) has different curricular provision, this study will consult with key stakeholders (pupils, teachers, parents) to test the acceptability of Y-PATH and adapt intervention content for delivery in NI post-primary schools.

**Methods:**

The development phase involved reviewing the NI PE curriculum, a cross-sectional online survey with NI pupils (n = 1541, 11-14 years), focus groups (n = 17) with pupils aged 11-16 years, and interviews with parents and teachers (n = 15). In addition, 22 pupils participated in a Youth Advisory Group and were asked to provide feedback on intervention resources and proposed data collection measures for future feasibility testing. Descriptive statistics and chi-square analyses were run for quantitative data. Qualitative data were analysed using thematic analysis, data were summarised, and themes were generated.

**Results:**

Participants reported wanting to take part in more physical activity with their friends, during school time and to be given a choice of activities in their PE classes. Parents in NI indicated that they thought the Y-PATH programme would be beneficial for children and reported that they would be willing to be involved with the parent component of the intervention, while teachers noted some concerns in relation to the additional workload the intervention might entail. Feedback from the Youth Advisory Group suggested proposed outcome measures were acceptable, with detailed feedback provided on the design and functionality of Y-PATH resources, for example, the student journal.

**Conclusions:**

The findings from this study suggest that the Y-PATH intervention would be suitable for schools in NI. The adapted Y-PATH NI programme will be tested in a feasibility trial and a process evaluation will determine the fidelity and acceptability of the Y-PATH intervention within the NI setting. These findings will help policymakers and researchers develop interventions that will be effective in increasing physical activity levels amongst youth.

**Support/Funding Source:**

This study is funded by Northern Ireland Chest Heart and Stroke.

